# Method to identify efficiently cleaved, membrane-bound, functional HIV-1 (Human Immunodeficiency Virus-1) envelopes

**DOI:** 10.1016/j.mex.2019.04.013

**Published:** 2019-04-17

**Authors:** Sweety Samal, Manish Bansal, Supratik Das

**Affiliations:** THSTI-IAVI HIV Vaccine Design Program, Translational Health Science and Technology Institute, Faridabad, 121001, Haryana, India

**Keywords:** Identifying cleaved, functional HIV-1 Envs, Biochemistry, Broadly neutralizing antibodies, Non-neutralizing antibodies, FACS, Pseudovirus neutralization, gp120 shedding, Biotinylation, Neutravidin-agarose pull down, Plasma membrane fraction, Immunoprecipitation

## Abstract

An ideal vaccine against HIV-1 will specifically elicit bNAbs (broadly neutralizing antibodies) which can cross-neutralize a wide spectrum of circulating viral strains belonging to different clades. The current paradigm for developing such a vaccine is to generate HIV-1 envelope (Env)-based immunogens which can specifically elicit bNAbs. For this purpose, it is necessary to identify Envs, belonging to different clades, suitable for immunogen design. Efficient cleavage of the HIV-1 Env precursor gp160 polypeptide into its constituent subunits determines its ability to selectively bind to bNAbs and poorly to non-NAbs (non-neutralizing antibodies), properties desirable in Env-based immunogens. Thus, efficiently cleaved HIV-1 Envs with desirable antigenic properties can be good candidates for developing immunogens. Here we describe in detail a six step method we have used in our laboratory to identify such efficiently cleaved Envs. Some of these protocols are optimizations of previously reported assays such as FACS-based cell surface antibody binding assay, pseudovirus neutralization assay and gp120 shedding assay. Other protocols like biotinylation-neutravidin-agarose pull-down assay and plasma membrane protein immunoprecipitation assay have been developed by taking inputs from reagent/kit manufacturer’s protocols and previous studies. These protocols will help the field in identifying more such Envs which can be used for immunogen development.

• Six step process to identify efficiently cleaved, membrane-bound, functional HIV-1 Envs with high degree of repeatability.
• Method applicable for characterizing any HIV-1 envelope protein.
• New method of immunoprecipitation of plasma membrane fraction to validate efficiently cleaved HIV-1 envelopes.

## Graphical abstract

**Figure:**
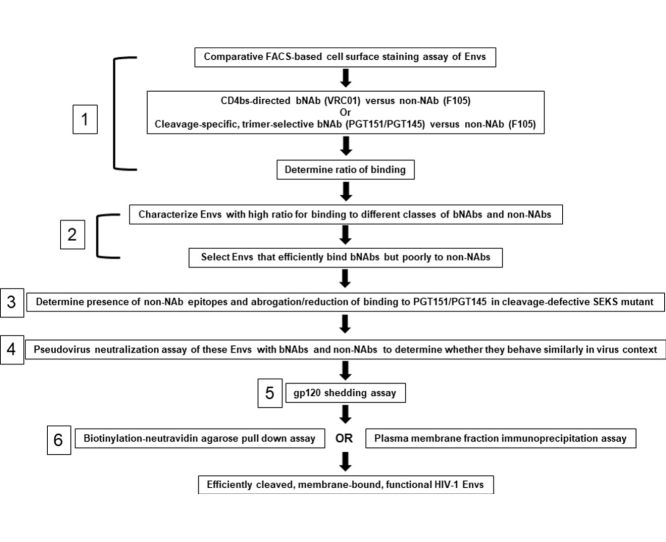

**Specifications Table**

**Table:** 

Subject area:	Immunology and Microbiology
More specific subject area:	HIV-1 Envelope
Method name:	Identifying cleaved, functional HIV-1 Envs
Name and reference of original method:	Adapted from: • Pancera M, Wyatt R (2005); Virology 332:145-156. • Li M, Gao F, Mascola JR et al (2005); J Virology 79(16):10108-10125. • Ringe R, Thakar M, Bhattacharya J. (2010); Retrovirology 7: 76. • Chakrabarti BK, Pancera M, Phogat S, et al (2011); AIDS Res Hum Retroviruses 27(8):877-87. • Boliar S, Das S, Bansal M, Shukla BN, Patil S, et al (2015); PLoS One 10: e0122443.
Resource availability:	• R-Phycoerythrin-conjugated AffinityPure F(ab’)_2_ Fragment Goat Anti-Human IgG, F(ab’)_2_ Fragment Specific (minimal cross-reaction to Bovine, Horse, and Mouse Serum Proteins); Cat# 109-116-097; Jackson ImmunoResearch Laboratories Inc. • FlowJo software, version 10.0.6, Tree Star Inc. • sCD4-183, Cat# 7356; sCD4, Cat# 4615; NIH AIDS Reagent Program • Dounce homogenizer; Cat# D8938; Sigma-Aldrich • Plasma membrane protein extraction kit; Cat# ab65400; Abcam • EZ-link Sulfo-NHS-LC-Biotin; Cat# 21335; Thermo Scientific • Neutravidin agarose; Cat#. 29204; Thermo Scientific • anti-gp120 Env (clade A, B, C) antibody; Cat# 5419, 5414, 5411; AbLinc.

## Materials

1 Amicon Ultra centrifugal filters (Ultracel – 30 K) (Millipore, Cat# UFC903096).
2 BD FACSFlow (BD Biosciences, Cat# 342003).
3 Bright-Glo Luciferase Assay System (Promega, Cat# E2620).
4 Britelite plus Luminescence Reporter Assay System (PerkinElmer, Cat# 6066769).
5 Bromophenol Blue sodium salt (SIGMA, Cat# B8026, electrophoresis/molecular biology grade).
6 Cesium chloride (ThermoFisher Scientific/Invitrogen, Cat# 15507-023, Ultrapure, optical grade).
7 DEAE Dextran (SIGMA, Cat# D9885).
8 DL-Dithiothreitol (SIGMA, Cat# 43819, 99%, RT).
9 DMEM (GIBCO, Cat# 11965-092).
10 EDTA (SIGMA, Cat# E5134, molecular biology grade).
11 Ethidium bromide (HIMEDIA, Cat# MB071, molecular biology grade).
12 Formaldehyde (SIGMA Cat# 47608, 36%, molecular biology grade).
13 FreeStyle 293 Expression Medium (GIBCO, Cat# 12338-018).
14 Fugene 6 transfection reagent (Promega, Cat# E2691).
15 Goat anti-rabbit secondary antibody-HRP conjugated (Santa Cruz Biotechnology, Cat# sc-2004).
16 Glutamax (GIBCO, Cat# 35050-061).
17 Glycerol (HIMEDIA, Cat # MB060, molecular biology grade).
18 Glycine (SIGMA, Cat# G8898, electrophoresis grade).
19 HCL (Honeywell Fluka, Cat# 84415, 37%, TraceSELECT, for trace analysis, fuming).
20 HI-FBS (Heat inactivated Fetal Bovine Serum) (GIBCO, Cat# 16140-071).
21 IGEPAL CA-630 (SIGMA, Cat# I8896, molecular biology grade).
22 Immobilized Protein A Resin/Protein A Agarose (G Biosciences, Cat# 786–824).
23 Isopropanol (Fisher Chemical, Cat# P/7500/17, AR grade).
24 Lectin from *Galanthus nivalis* (snowdrop) (SIGMA, Cat# L8275).
25 Methanol (Avantor, Cat# 9830-03, LC–MS reagent).
26 Nuclease-free water (HIMEDIA, Cat# ML024, molecular biology grade).
27 OptiMEM (GIBCO, Cat# 31985-070).
28 PEI MAX (MW 40 K) (Polysciences Inc., Cat# 24765, transfection grade).
29 Penn Strep (penicillin-streptomycin) (GIBCO, Cat# 15140-122).
30 Pierce Protease inhibitors, EDTA-free (ThermoFisher Scientific, Cat# 88666).
31 Pierce BCA Protein Assay Kit (ThermoFisher Scientific, Cat# 23227).
32 PBS (Phosphate buffered saline) (HIMEDIA, Cat# TL1006, TC grade w/o calcium/magnesium).
33 Polystyrene Round-bottom tube (FALCON, Cat# 352052).
34 Protein G agarose (InvivoGen, Cat# gel-agg-5).
35 Quick-Seal Polyallomer tube (5.1 ml, 13 × 51 mm) (Beckman Coulter, Cat # 342412).
36 Sodium deoxycholate (SIGMA, Cat# D6750, 97% [titration]).
37 Sodium acetate (SIGMA, Cat# S2889, molecular biology grade).
38 SDS (SIGMA, Cat# L3771, molecular biology grade).
39 Sodium chloride (HIMEDIA, Cat# MB023, AR grade).
40 SuperSignal West Dura Extended Duration Substrate (ThermoFisher Scientific, Cat# 34075).
41 TMB solution (ThermoFisher Scientific, Cat# 002023).
42 Tris free base (HIMEDIA, Cat# MB029, molecular biology grade).
43 Triton X-100 (SIGMA, Cat# T8787, molecular biology grade).
44 TrypLE Express (GIBCO, Cat# 12605-010).
45 Tween-20 (HIMEDIA, Cat# TC287, cell culture grade).
46 96-well white solid optic plates (PerkinElmer, Cat# 6005290).
47 2-Mercaptoethanol (SIGMA, Cat# M6250, electrophoresis grade).
48 1-Butanol (SIGMA, Cat# 360465, ACS grade).

## Method summary

The HIV-1 envelope (Env) glycoprotein is the sole target of broadly neutralizing antibodies (bNAbs). These antibodies develop in a fraction of HIV-1 infected patients and show potent and broad cross-clade neutralization. One of the strategies for developing a vaccine against HIV-1 is to use Env-based immunogens to immunize vaccinees that will result in activation of the humoral immune response and elicitation of bNAbs. The Env-based immunogens should specifically elicit bNAbs and not non-NAbs (non-neutralizing antibodies) and therefore they should specifically bind to bNAbs with high efficiency and weakly to non-NAbs. The HIV-1 Env is a trimer of a heterodimer of the gp120 soluble and gp41 transmembrane subunits that is formed by proteolytic cleavage of the precursor gp160 polypeptide. Previously, it has been shown that efficient cleavage of the Env gp160 precursor into its constituent subunits co-relate with specific binding to bNAbs and weak binding to non-NAbs [1,2], properties essential in functional Envs suitable for vaccine immunogen design. Based on these studies a six step strategy (see below) was devised to identify naturally occurring, membrane-bound, efficiently cleaved Envs which show antigenic properties suitable for immunogen design.

1 Screening of HIV-1 Envs for their differential binding to CD4bs-directed bNAb VRC01 or cleavage-specific, trimer-selective bNAbs PGT151/PGT145 versus non-NAb F105 by FACS-based cell surface antibody binding assay. The ratio of binding to bNAb as compared to non-NAb is determined from MFI (mean fluorescent intensity) values. The Envs that show highest ratio of binding (typically more than 2.0) are chosen for further characterization.
2 The chosen Envs are then tested in FACS-based cell surface antibody binding assays for their ability to bind a panel of bNAbs and non-NAbs. Envs that bind efficiently to different bNAbs but weakly to non-NAbs are taken up for further characterization.
3 That the weak binding to non-NAbs is not due to a lack of epitopes is verified by testing the cleavage-defective REKR (cleavage site) to SEKS mutant in FACS-based cell surface antibody binding assay against a panel of non-NAbs. The SEKS mutant binds non-NAbs with higher efficiency than wild-type if the epitopes are present. In addition, binding of the SEKS mutant to the cleavage-specific, trimer-selective bNAbs PGT151/PGT145 is tested against wild-type Env. SEKS mutants of efficiently cleaved Envs show reduced binding to these bNAbs.
4 The selected Envs are also tested against a panel of bNAbs and non-NAbs for their ability to be neutralized by these antibodies in pseudovirus neutralization assay and the IC_50_ values are determined. Efficiently cleaved Envs are efficiently neutralized by bNAbs (low IC_50_ values) while they are not neutralized or weakly neutralized by non-NAbs (high IC_50_ values).
5 The gp120-gp41 association in the Env trimer is labile leading to spontaneous shedding of gp120 which can be measured by ELISA. Furthermore, sCD4 binds to Env and causes conformational changes which lead to induced shedding of gp120. So the gp120 shedding assay is used as a measure of efficient cleavage of Envs on the cell surface.
6 Finally, direct observation of efficient cleavage of Envs can be made by either biotinylating the cell surface proteins of Env transfected cells followed by neutravidin-agarose pull-down and then bead bound proteins visualized by western blot analysis to determine whether they show gp160 band (uncleaved) or gp120 band (cleaved); or by isolation of plasma membrane fraction of Env transfected cells followed by immunoprecipitation with cleavage non-specific bNAbs like VRC01 and western blot analysis to determine gp160 or gp120 bands.

Here we describe in detail the experimental protocols used in this six step method to identify naturally occurring, membrane-bound, efficiently cleaved Envs. The cesium chloride method for plasmid DNA preparation is derived from standard laboratory procedure. The human monoclonal antibody preparation and purification method from 293 F cells has been developed based on routine procedure of transfection and protein preparation from this type of cell line and antibody purification. Some of the methods are optimizations of previously published experimental procedures e.g. FACS-based cell surface antibody binding assay [1,3], pseudovirus neutralization assay [4,5] and gp120 shedding assay [3]. The biotinylation followed by neutravidin-agarose pull-down assay has been developed based on the manufacturer’s protocol (Thermo Scientific, Cat# 21335, 29204) and similar previously published assays [1,6]. The plasma membrane protein immunoprecipitation assay has been developed based on the manufacturer’s protocol for isolation of the plasma membrane fraction (Abcam, Cat# ab65400). Using this six step process we have identified the naturally occurring, membrane-bound efficiently cleaved Envs A5 and BG505 (clade A), JRCSF (clade B), 4-2.J41 (clade C) and LT5.J4b12C (clade B/C) [[6], [7], [8], [9]].

## Method details

### Preparation of human mAbs (monoclonal antibodies) against HIV-1 Env (envelope)

**Procedure**

All plasmid DNAs used for transfection were prepared by cesium chloride method.

1 Crude plasmid DNA in 3 ml of TE (Tris-EDTA) (pH 8.0) buffer was prepared by alkaline lysis method as described in ‘Molecular Cloning; A Laboratory Manual (Fourth Edition) by Michael Green and Joseph Sambrook, Chapter 1, Protocol 2′. Next, 4.5 g of cesium chloride and 100 μl of ethidium bromide solution (10 mg/ml) were added to a 15 ml falcon tube. Plasmid DNA in TE was added to the tube and mixed thoroughly. The mixture was transferred to a Beckman Polyallomer tube with the help of a syringe, the tubes were sealed with a sealer and weighed (weight should be around 9.9–10 g). The tubes were centrifuged in an Optima XE-100 Beckman Coulter ultracentrifuge (NVT 100 rotor) at 227,000 × *g* and 22 °C for 18–22 h. The closed circular and nicked/linear plasmids were discernible as rings from bottom to top, respectively. The centrifuge tube was unsealed and closed circular DNA was collected by puncturing the tube with a 10 cc syringe fitted with an 18 gauge needle just below the DNA band and drawing the band slowly into the syringe. For better visualization of the bands longwave UV light may be used. Ethidium bromide was removed from the DNA by extracting six times with an equal volume of water saturated n-butyl alcohol (or until pink color is gone). Then 3 M sodium acetate pH (5.0) was added to get final concentration of 0.3 M and the tube inverted 4–5 times. Next, isopropanol (0.7 × total volume) was added to precipitate DNA and centrifuged at 3214 × *g* and 4 °C for 30 min in a table-top centrifuge. The supernatant was discarded, pellet was washed with 70% ethanol and air dried. Finally, the pellet was dissolved in nuclease-free water.
2 293 F cells were split one day before transfection and 1.2 × 10^6^ cells/ml seeded in 500 ml of FreeStyle 293 Expression medium. Suspension cultures were grown in a CO_2_ shaker-incubator. Next day, two 50 ml Falcon tubes were prepared and 20 ml of OptiMEM (reduced serum medium), pre-warmed to 37 °C was added to each tube. 800 μg of plasmid DNA expressing antibody heavy chain and 400 μg of plasmid DNA expressing antibody light chain were added to tube A. 4 ml of PEI MAX (Linear Polyethylenimine Hydrochloride, MW 40 K) (1 mg/ml stock) was added to tube B and both tubes were incubated for 5 min at RT (room temperature). The contents of tube A were added to tube B, mixed slowly but thoroughly by inverting the tube and incubated for 30 min at RT. The contents of the tube were added to the flask containing 293 F cell suspension culture. Next day, 500 ml of Freestyle media was add to the flask and grown for 5 days.
3 The cells were pelleted by centrifugation at 13,750 × *g* and 4 °C for 30 min in a Sorvall RC6+ centrifuge (ThermoScientific) and the supernatant was collected. The supernatant was stored at −80 °C. On the day of purification, supernatant was thawed and passed through a Protein A agarose column (4 ml of beads) pre-washed with ice-cold PBS (phosphate buffered saline). The column was washed with 40–80 ml of ice-cold PBS. Elution was carried out with 10–15 ml of elution buffer (100 mM glycine-HCl, pH 2.5), and the eluate neutralized with 1 M Tris−HCl, pH 8.5 and dialyzed overnight (O/N) against 1 L of ice-cold PBS. The antibody solution was concentrated with Amicon Ultra centrifugal filters (Ultracel – 30 K), the protein concentration was determined and antibody preparation was stored at – 80 °C in aliquots.

### FACS (fluorescence-activated cell sorting)-based cell surface antibody binding assay

The relatively superior binding of HIV-1 Envs to bNAbs in comparison to non-NAbs is a good measure of potential cleavability of an Env [1,[6], [7], [8]]. This protocol is an adaptation of a previously described assay [1,3]. The number of cells to be used for the assay after transfection, wash conditions for harvesting and antibody incubation, time of incubation and data collection parameters in the FACS analyzer were optimized. JRFL (clade B) and REKR to SEKS cleavage-defective mutants of Envs were used as controls.

**Procedure**

1 10 × 10^6^ 293 T cells were plated in complete DMEM medium (DMEM [Dulbecco’s Modified Eagle’s medium], 10% HI-FBS [Heat-Inactivated Fetal Bovine Serum], 1X Penn-Strep [penicillin-streptomycin], 1X Glutamax [L-alanyl-l-glutamine dipeptide in 0.85% NaCl]) in 150 mm cell culture plates one day prior to transfection.
2 For Env expression under the HIV-1 LTR (long terminal repeat) promoter from the *env*+ pSVIIIenv plasmid [10], co-transfect pc-tat plasmid (expresses Tat required for expression of Env under the control of the viral LTR promoter) [11] in the ratio of Env:Tat of 20:1. OptiMEM, pre-warmed to 37 °C, was added to two separate eppendorf tubes in the volume of 0.9 ml to eppendorf tube 1 and 100 μl to eppendorf tube 2. Then 30–50 μg (maximum) of plasmid DNA (*env*+ pSVIIIenv or *env*+ pcDNA 3.1/ expression vector) was added to eppendorf tube 2 and 3x volume of Fugene 6 transfection reagent (Invitrogen) was added to eppendorf tube 1 and incubated for 5 min at RT. The contents of eppendorf tube 2 was added to eppendorf tube 1 and mixed up and down three times with pipette. The contents of the tube were mixed slowly but thoroughly by inverting the tube and incubated for 30 min at RT. The mixture was added to cells by spreading drops throughout the plate and incubated at 37 °C in CO_2_ incubator O/N. Next day, the medium was changed with fresh complete DMEM medium and incubated O/N in CO_2_ incubator.
3 After 36–48 h of transfection the medium was removed and cells were harvested by adding 5 ml of pre-warmed 5 mM EDTA (Ethylenediaminetetraacetic acid disodium salt dehydrate) in PBS and incubated at RT or 37 °C until cells start coming off. The cells were collected in 5 ml of buffer 1 (DMEM + 5% HI-FBS), mixed and centrifuged at 475 × *g* and 22 °C for 4 min in a table-top centrifuge. The supernatant was discarded. The cells were washed with 10 ml of buffer 1 twice by mixing and centrifugation. The cell pellet was resuspended in 2 ml of buffer 1 and mixed thoroughly. The cells were counted using a hemocytometer.
4 Approximately 2.5 × 10^5^ cells were added to each well of a 96 well U-bottom plate. Buffer 1 was added upto 200 μl using multi-channel pipette and the plate centrifuged at 475 × *g* and 22 °C for 4 min in a table-top centrifuge. The supernatant was aspirated and the cell pellet was left wet.
5 Primary antibodies were diluted in buffer 1 to keep the highest concentration at 20 μg/ml. The antibodies were serially diluted (two fold) in buffer 1 keeping the concentration at 10, 5, 2.5, 1.25 μg/ml, respectively. Each antibody solution (100 μl) was added to wells of the plate in duplicate starting from 0 μg/ml (no primary antibody control) upto 20 μg/ml, consecutively. The contents of each well were mixed up and down 6–8 times from the no to highest antibody concentration well using a multi-channel pipette and then incubated for 1 h at RT.
6 Buffer 1 (100 μl) was then added to each well and centrifuged at 475 × *g* and 22 °C for 4 min in a table-top centrifuge. The supernatant was aspirated keeping the cell pellet wet. The washing step was repeated two more times with 200 μl of buffer 1.
7 Secondary antibody (R-Phycoerythrin-conjugated AffinityPure F(ab’)_2_ Fragment Goat Anti-Human IgG) was diluted in buffer 1 (1:200 dilution) and mixed well. Secondary antibody in buffer 1 (100 μl) was added to each well and mixed by pipetting up and down 6–8 times with multi-channel pipette from no to highest antibody concentration. The mixture was incubated for 45 min to 1 h at RT in the dark (plate wrapped in aluminium foil).
8 Buffer 2 (PBS + 5% HI-FBS) (100 μl) was added to each well of plate and plate centrifuged at 475 × *g* and 22 °C for 4 min. The supernatant was removed by aspiration and the cell pellet was left wet. The washing step was repeated with 200 μl of buffer 2 two more times. The cells were fixed with 200 μl of 0.5% formaldehyde in buffer 2. The plate was wrapped in aluminium foil and stored at 4 °C.
9 The stained cells from each well of the plate were transferred to a 5 ml polystyrene round-bottom tube and the cells analyzed by FACS Canto II analyzer (BD Biosciences). Reading is taken in the PE (R-phycoerythrin) channel. The voltages for FSC (forward scatter), SSC (side scatter) and PE (R-phycoerythrin) were adjusted such that size and fluorescence intensity were properly visualized on the plots on the screen. 15,000–20,000 cells were acquired per well. BD FACSFlow buffer was used as the sheath fluid. The data were analyzed using the FlowJo software by gating cells and avoiding debris and determining the mean intensity of PE. MFIs (mean fluorescent intensity) were then plotted against primary antibody concentrations to get binding curves. Ratio of binding to bNAbs versus non-NAbs was determined by taking the MFIs at 20 μg/ml primary antibody concentration or by taking the AUC (area under curve) of the binding curves.

**Fig. 1:**
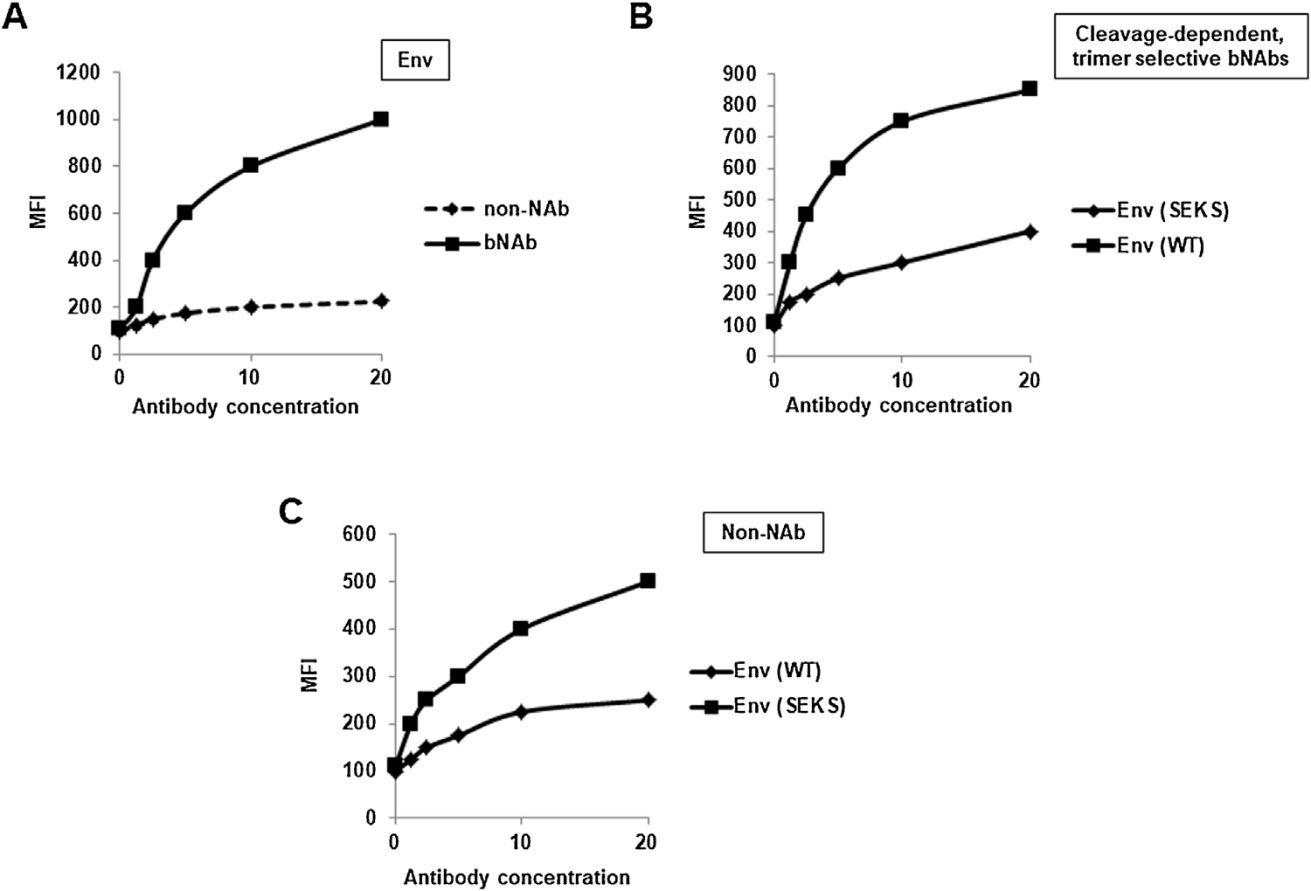
Schematic representation of FACS-based cell surface antibody binding assay of efficiently cleaved Envs with different monoclonal antibodies, (A) Env binding to bNAb versus non-NAb, (B) wild type Env versus cleavage-defective SEKS mutant binding to cleavage-dependent, trimer-selective bNAbs e.g. PGT151, PGT145, (C) wild type Env versus SEKS mutant binding to non-NAbs.

Schematic representation of typical experiments using this method is shown in Fig. 1 and actual experimental results can be found in Refs. [1,3,[6], [7], [8]]. In general we have observed that efficiently cleaved Envs bind strongly to majority of bNAbs and weakly to non-NAbs (Fig. 1A and Refs. [1,3,[6], [7], [8]]). Mutation of cleavage site SEKS to REKR (cleavage-defective) causes reduction in binding to trimer-selective, cleavage-specific bNAbs like PGT145 and PGT151 (Fig. 1B and Refs. [7,8]). Presence of non-NAb epitopes in the Env can be checked using the cleavage-defective SEKS mutant which exposes non-NAb-epitopes leading to greater binding than wild-type (Fig. 1C and [3,[6], [7], [8]]).

### Pseudovirus neutralization assay using TZM-bl cells

The TZM-bl assay is a standard neutralization assay that measures antibody-mediated neutralization of HIV- 1 envelopes [4,5]. After a single round of infection with Env-pseudotyped viruses, the reduction in HIV-1 Tat-regulated firefly luciferase (Luc) reporter gene expression is evaluated in the absence or presence of antibody. The cell seeding density, 293 T cells passage number (restricted to 20 passage), incubation temperature with antibody, tissue culture infectivity dose (TCID_50_) of pseudoviruses titration were optimized.

**Procedure**

1 Pseudotyped viruses carrying patient Env were produced by cotransfection of either *env*+ pSVIIIenv or *env*+ pcDNA 3.1/ expression vector and *env*-deleted HIV-1 backbone vector (pSG3ΔEnv) (Env: Backbone vector 1:2), into 293 T cells with 70–80% confluence in 6-well tissue culture trays using Fugene 6 transfection reagent (DNA: Fugene 6 - 1:3).
2 Cell supernatants carrying progeny pseudotyped viruses were harvested at 48 h post-transfection, centrifuged at 475 × *g* for 10 min. in a table-top centrifuge. The supernatant was stored at −80 °C until further usage.
3 The infectivity assays were done in TZM-bl cells in 96-well microtiter plate as described elsewhere [4,5]. Infectivity titers were determined by measuring the luciferase activity (see below except that there was no antibody incubation) as described [4,5].
4 Pseudoviruses containing desired envelope (50 μl equivalent to 1 × 10^5^ RLU (relative luciferase unit) were incubated with serial-fold dilutions of monoclonal antibodies in duplicate in a total volume of 100 μl for 1 h at 25 °C in 96-well flat-bottom plates.
5 Freshly trypsinized TZM-bl cells (10,000 cells) in 100 μl of DMEM containing 25 μg/ml DEAE-Dextran were added to each well containing pseudovirus and antibody. One set of control wells received cells plus pseudovirus (virus control) only while another set of wells received cells plus medium (background control).
6 After 48 h of incubation, luciferase activity was measured by using the Bright-Glo Luciferase Assay System (or britelite plus Luminescence Reporter Assay System). 80 μl of sample were discarded and then 40 μl of Bright-Glo Luciferase substrate was added and kept at RT for 2 min of incubation to allow cell lysis. After incubation, 130 μl of cell lysates were transferred to 96-well white solid optic plates for measurements of luminescence using a Victor 2 Luminometer (Perkin-Elmer Life Sciences).
7 The 50% inhibitory concentration (IC_50_) is defined as the sample concentration (in the case of sCD4 [soluble CD4] and mAbs) that causes a 50% reduction in relative luminescence units (RLU) as compared to the virus control after subtraction of background RLU.

**Fig. 2:**
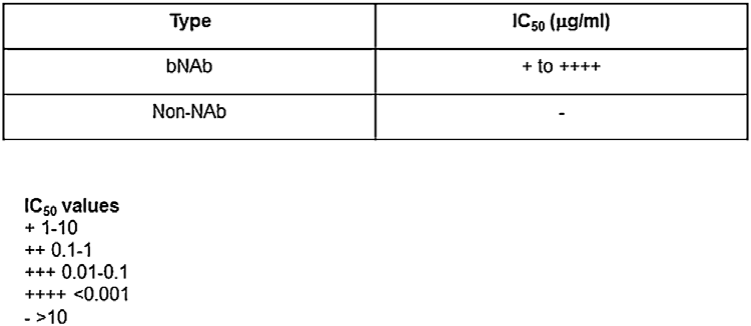
Schematic table of typical pseudovirus neutralization assay experiments (IC_50_ values) of efficiently cleaved Envs with bNAbs versus non-NAbs. IC_50_ values less than 1 suggests potent neutralization and greater than 10 suggests weak neutralization.

Schematic representation of typical experiments using this method is shown in Fig. 2 and actual experimental results can be found in Refs. [1,6,8]. Efficiently cleaved Envs are potently neutralized by a majority of bNAbs with low IC_50_ values while they are weakly neutralized by non-NAbs with IC_50_ values higher than 10 (Fig. 2 and [1,6,8]).

### gp120 shedding assay

This protocol is adapted from a previously described assay [3]. The number of cells to be transfected, lectin concentration, incubation volume with and without sCD4 and condition, centrifugation and incubation condition, time of incubation and wash volumes and rabbit primary antibodies used and their dilution were optimized.

**Procedure**

1 293 T cells in one 150 mm plate per reaction were transfected as described above (1.2). Mock transfected cells were used as a negative control. Cells were harvested as described above (1.2). The cells were re-suspended in 1 ml of buffer 2 and 50 μg/ml of sCD4 was added to tube designated w/ sCD4. The cells were mixed and incubated for 1 h at 4 °C with repeated inverting of tube (every 5 min). The cells were centrifuged at 400 × *g* for 5 min at 4 °C in a microfuge. The pellet was discarded and the supernatant re-centrifuged at 20,000 × *g* for 10 min at 4 °C in a microfuge. The supernatant was collected and stored at −80 °C.
2 A 96 well flat-bottom plate was taken, 100 μl of lectin (final concentration 2.5 μg/ml in PBS) was added to each well and incubated O/N at 4 °C. The liquid was discarded, 100 μl of blocking buffer (buffer 2 + 5% dry milk, mixed thoroughly) was added and incubated for 1 h at RT with gentle shaking. The liquid was discarded and the wells were washed three times with wash buffer PBST (PBS + 0.1% Tween-20) by adding 120 μl of washing buffer with multi-channel pipette and discarding liquid. The supernatant was serially diluted (1:3) in buffer 2 upto 1:729. Buffer 2 and mock transfected supernatant were taken as controls. The undiluted, serially diluted and control supernatants (100 μl each) were added to each well of the plate in triplicate, and incubated for 1 h at RT with gentle shaking. The wells were washed as above.
3 To each well 100 μl of antibody solution [rabbit anti-Env polyclonal antibody at 1:1000 dilution in incubation buffer (PBS + 1:10 dilution of blocking buffer, mixed thoroughly)] was added and incubated for 1 h at RT with gentle shaking. Wells were washed as above. Next, 100 μl of goat anti-rabbit secondary antibody-HRP (Horseradish Peroxidase) conjugated at 1:1000 dilution in incubation buffer was added to each well. Incubated and washed as above.
4 After final wash, 100 μl of TMB (3,3′, 5,5;-TetraMethylBenzidine) solution was added and incubated at RT till blue color appears. Next, 100 μl of 1 N HCl (hydrochloric acid) was added and the color turns yellow. Colorimetric intensity reading was taken at 450 nm using a plate reader (SPECTRAmax PLUS, Molecular Devices). The reading of buffer alone was subtracted from experimental readings and OD_450_ (Optical density measured at 450 nm wavelength) plotted against dilution.

**Fig. 3:**
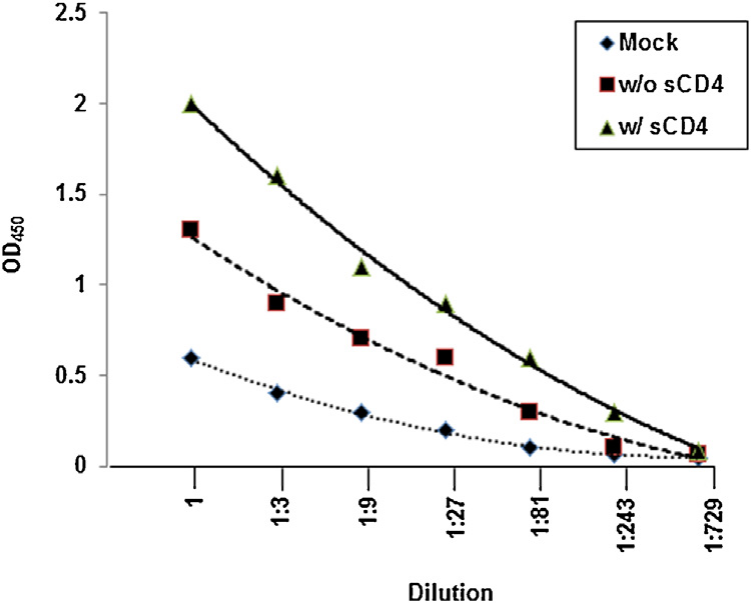
Schematic representation of typical gp120 shedding assay experiments (OD_450_ values) of efficiently cleaved Envs with mock transfected, Env-transfected without and with sCD4 incubation at different dilutions.

Schematic representation of typical experiments using this method is shown in Fig. 3 and actual experimental results can be found in Refs. [3,[6], [7], [8]]. Efficiently cleaved Envs shed gp120 subunit spontaneously due to the labile nature of gp120-gp41 association which can be further stimulated by incubation with sCD4 (Fig. 3 and Refs. [3,[6], [7], [8]]).

### Biotinylation and neutravidin-agarose pull-down assay

This protocol has been developed based on the manufacturer’s protocol (Thermo Scientific, Cat# 21335, 29204) and similar previously published assays [1,6]. We have optimized almost the entire protocol by ourselves except for wash buffer, concentration of biotin used and quenching buffer which were taken from the manufacturer’s protocol.

**Procedure**

1 One 100 mm plate of 293 T cells per sample were seeded such that at time of transfection next day plates were 70–80% confluent. The cells were co-transfected with plasmids expressing Envs either alone or with pc-tat plasmid (for Tat-dependent expression) expressing Tat protein (Env: Tat - 20:1). 36–48 h post transfection the cells were harvested and washed with PBS (pH 8).
2 The cells were labeled with freshly prepared 2 mM biotin (EZ-link Sulfo-NHS-LC-Biotin) in PBS (pH 8) for 30 min at 4 °C, washed with PBS twice (centrifuged at 475 x g in a table-top centrifuge), incubated with 50 mM glycine in PBS (pH 7.5) for 30 min at 4 °C with rotation and then washed twice with PBS (centrifuged at 475 × *g* in a table-top centrifuge).
3 Cell pellet was resuspended in RIPA (Radioimmunoprecipitation assay) buffer (50 mM Tris−HCI pH 7.4, 1% IGEPAL, 0.5% sodium-deoxycholate, 0.1% SDS, 150 mM sodium chloride, 2 mM EDTA and protease inhibitors) and incubated on ice for 30 min.
4 The cell extract was centrifuged at 16,000 × *g* for 30 min at 4 °C in a microfuge and the supernatant was subjected to precipitation with 100 μl slurry of high capacity neutravidin agarose (pre-washed with PBS) for 2 h at RT with rotation.
5 Finally, beads were washed three times with PBS + 1% Triton X-100 (centrifuged at 400 × *g* and 4 °C in a microfuge) and washed beads were resuspended in 50 μl of 1x SDS-PAGE (sodium dodecyl sulfate-polyacrylamide gel electrophoresis) loading buffer [50 mM Tris−HCl (pH 6.8), 2% (w/v) SDS, 715 mM 2-mercaptoethanol, 0.1% (w/v) bromophenol blue, 10% (v/v) glycerol), subjected to western blot analysis [transfer polypeptides from SDS-PAGE gels to PVDF membrane in transfer buffer (25 mM Tris, 192 mM glycine, 20% methanol v/v) at 250 mA for 1 h under ice-cold condition] using 1:1000 dilution of rabbit anti-Env (gp120) polyclonal antibodies as probes (O/N incubation with antibodies in cold room with gentle mixing, washed three times with TBST [20 mM Tris−HCl (pH 7.4), 150 mM sodium chloride, 0.1% Tween-20] followed by incubation with 1:1000 dilution of goat anti-rabbit secondary antibody-HRP conjugated at RT for 1 h, washed three times with TBST and blot developed using SuperSignal West Dura Extended Duration Substrate).

**Fig. 4:**
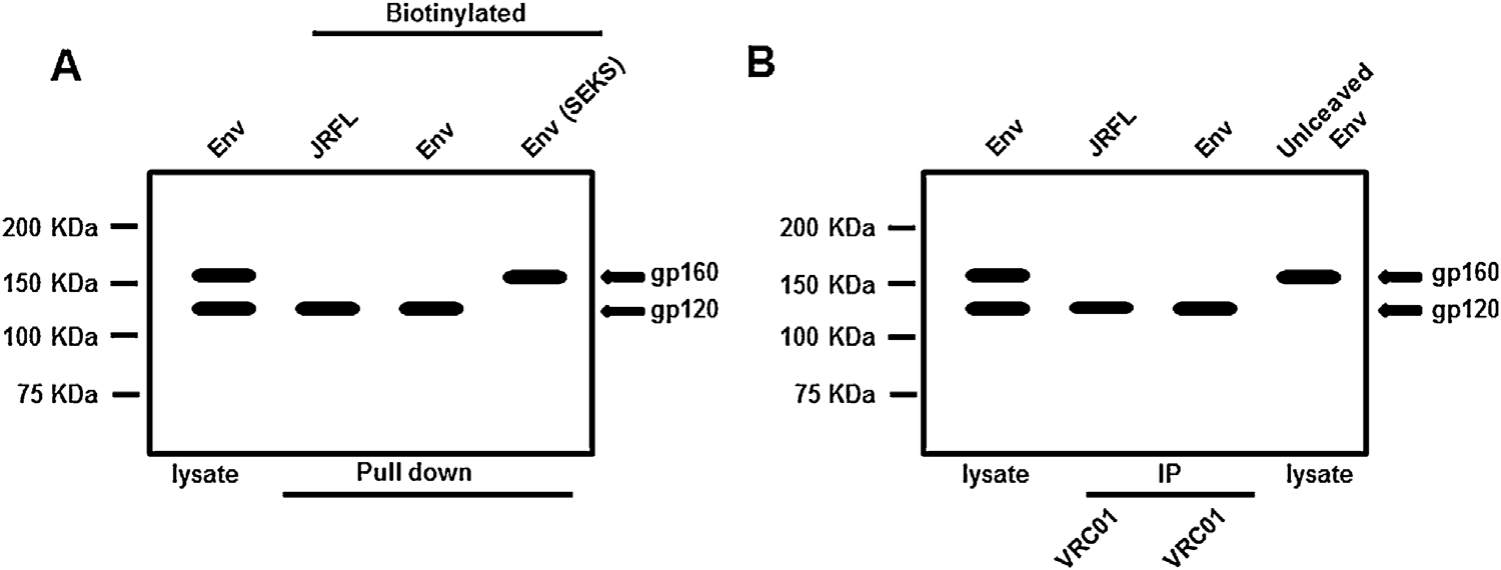
(A) Schematic representation of typical experiments showing western blot analysis of neutravidin agarose precipitates of cell surface biotinylated, efficiently cleaved, Env proteins with controls as shown, (B) schematic representation of typical experiments showing western blot analysis of immunoprecipitates of efficiently cleaved Env proteins from isolated plasma membrane fractions with cleavage non-specific bNAb VRC01 and controls as shown.

Schematic representation of typical experiments using this method is shown in Fig. 4A and actual experimental results can be found in Ref. [12]. In this assay, following western blot of neutravidin agarose pull-down of biotinylated cell surface proteins, efficiently cleaved Envs will show only gp120 band (Fig. 4A and [12]) whereas partially cleaved Envs will show both gp120 and gp160 bands and uncleaved Envs primarily gp160 band (Fig. 4A). Uncleaved Env SEKS mutant will show only gp160 band (Fig. 4A and [12]) and the well-known cleaved Env JRFL will show only gp120 band (Fig. 4A).

### Plasma membrane protein immunoprecipitation assay

This protocol has been developed and optimized by ourselves except for isolation of plasma membrane fraction which was taken from the manufacturer’s protocol (Abcam, Cat# ab65400) and the homogenization and the mixing of upper and lower phase solutions for plasma membrane fraction extraction were optimized.

**Procedure**

1 293 T cells were transfected, harvested and washed as described above (1.2). For transfection of each Env, three 150 mm plates were used to obtain enough cells for further processing. For harvesting, buffer 1 was used and for washing, buffer 2 was used. Optionally, cells were finally washed once with homogenization buffer from Plasma membrane protein extraction kit.
2 Cell pellet was re-suspended in 1 ml of ice-cold homogenization buffer (containing protease inhibitors from kit) and transferred to dounce homogenizer (2 ml working volume). The cells were homogenized with 50 strokes of pestle A followed by 20 strokes of pestle B.
3 The homogenized cell mixture was transferred to eppendorf tube (1.5 ml) and centrifuge at 700 × *g* for 10 min at 4 °C in a microfuge. The supernatant was removed and the pellet discarded. The supernatant was re-centrifuged as above; the supernatant was collected and centrifuged at 10,000 × *g* for 30 min at 4 °C. The supernatant was discarded. The pellet contains total cellular membrane proteins.
4 The pellet was re-suspended in 200 μl of upper phase solution by mixing up and down with pipette and gentle vortexing 6–8 times. 200 μl of lower phase solution was added. Mixed by vortexing 50 times (30 s each) and then incubated on ice for 5–10 min. The mixture was centrifuged at 1000 × *g* for 5 min at 4 °C in microfuge. The upper phase (clear liquid) was gently removed and transferred to a fresh eppendorf tube. Fresh 100 μl of upper phase solution was added to the previous tube containing mixture and vortexed to mix and centrifuged as above. The upper phase (clear liquid) was gently removed and transferred to the second tube (total volume of extracted solution about 300 μl).
5 The extracted upper phase solution (about 300 μl) was diluted with ice-cold milli-Q water upto 1.5 ml of volume, mixed gently and incubated on ice for 5–10 min. The solution was centrifuged at 20,000 × *g* for 30 min at 4 °C in a microfuge. The supernatant was gently discarded and the pellet re-suspended in 100 μl of buffer 3 ([20 mM Tris−HCL (pH 7.5), 150 mM sodium chloride, 1 mM DTT, 1% Triton X-100, protease inhibitors] or [PBS, 1% Triton X-100, protease inhibitors]) by vortexing to get the plasma membrane fraction.
6 The protein concentration of the isolated plasma membrane fraction was determined with the Pierce BCA Protein Assay Kit using a microplate according to the manufacturer’s protocol. Equal amount of protein was used for immunoprecipitation by each antibody. Wherever comparative immunoprecipitation studies with different antibodies of the same Env were carried out which requires more protein, the isolated plasma membrane fractions were pooled and mixed and then divided into aliquots. Buffer 3 was added to each reaction to make volume upto 500 μl. 2 μg of mAb for each reaction was added and incubated O/N at 4 °C with mixing. Next day, 80 μl of PBS-washed Protein G agarose beads was added to each reaction and incubated for 1 h at 4 °C with mixing. The mixture was centrifuged at 400 × *g* for 5 min at 4 °C in a microfuge. The supernatant was discarded and the beads washed three times with PBS containing 1% Triton X-100 (5 min mixing followed by 5 min centrifugation as described above). All liquid was gently removed with gel loading pipette tip after final wash.
7 The washed protein-antibody complex-bound beads were re-suspended in 50 μl of 1x SDS-PAGE loading buffer and subjected to western blot analysis using rabbit anti-Env (gp120) polyclonal antibodies as probes as described above (1.5).

Schematic representation of typical experiments using this method is shown in Fig. 4B and actual experimental results can be found in Refs. [[6], [7], [8]]. An efficiently cleaved Env should show only gp120 band in western blots of immunoprecipitates of plasma membrane fractions of transfected cells with cleavage independent bNAbs like VRC01 that migrates faster than the gp160 band in uncleaved or partially cleaved Envs (Fig. 4B and Refs. [[6], [7], [8]]). Control JRFL Env, which is known to be efficiently cleaved, when immunoprecipitated with VRC01, show only gp120 band (Fig. 4B and Refs. [[6], [7], [8]]). Uncleaved control Env lysate should show only gp160 band (Fig. 4B and Refs. [7,8]).

## Discussion

HIV-1 viral diversity makes choosing envelope (Env) proteins suitable for immunogen design challenging. Due to this enormous diversity it is likely that a polyvalent vaccine will be necessary to tackle HIV-1 infection effectively. For this purpose, it is necessary to identify HIV-1 Envs suitable for immunogen design from different clades. Specific binding to bNAbs and weak binding to non-NAbs are desirable properties in HIV-1 Envs suitable for immunogen design as the purpose of a vaccine will be to specifically elicit bNAbs and not non-NAbs. Efficient cleavage of HIV-1 Env gp160 precursor polypeptide into its constituent subunits leads to formation of functional Envs which is co-related with their efficient binding to bNAbs and weak binding to non-NAbs. Thus, such efficiently cleaved Envs are suitable for vaccine immunogen design. In order to identify such Envs a six step method has been developed in our laboratory - the detailed protocols of which we report here. Some of these protocols comprise modifications and optimizations of earlier reported assays while others have been developed in our laboratory. These protocols will aid the field in the discovery of other such efficiently cleaved Envs with desirable antigenic properties and add to the repertoire of Envs that can be taken up for immunogen design.

## Additional information

For all assays, transfections were carried out using plasmid DNA prepared by cesium chloride method for best results. Some antibodies are expressed better than others. Volume of 293 F cells to be transfected should be adjusted according to obtained yield of antibody. We have observed that MFI of antibody binding to Envs in the FACS assay varies with time and condition of antibody storage. So it is necessary to use antibodies prepared together and stored similarly for comparative antibody binding studies of Envs. Antibodies should be prepared similarly for pseudovirus neutralization assay. For FACS assay cells should not be clumped, should not become dry or dead and liquid remaining after aspiration should be similar for best results. Buffers used for detaching and washing cells for FACS assay should be pre-warmed. It is advisable to carry out the gp120 shedding assay in quintuplicate as sometimes presence of cell debris or particles will affect the accuracy of readings in a well or two. Buffers used for detaching and washing cells in shedding assay should be pre-warmed. Buffer used for sCD4 incubation should be cooled to 4 °C. Buffers used for gp120 shedding assay should be at RT. For plasma membrane fraction immunoprecipitation experiments we use the well-established efficiently cleaved, functional Env JRFL as a positive control as it shows a single gp120 band in western blot. The cleavage-defective SEKS mutants often show anomalous migration or non-specific bands after immunoprecipitation of plasma membrane fractions making it difficult to use them as controls. Alternative controls to show gp160 bands are Envs that show both gp160 and gp120 bands in western blots or uncleaved Envs which show only gp160 band in western blots. Sometimes in immunoprecipitation experiments of plasma membrane fractions of Envs bands migrating faster than gp120 appear which may be due to differential glycosylation. Biotinylation followed by neutravidin-agarose pull-down is a more challenging experiment to execute than plasma membrane fraction immunoprecipitation.
